# Continuous Wet Spinning of Regenerated Silk Fibers from Spinning Dopes Containing 4% Fibroin Protein

**DOI:** 10.3390/ijms241713492

**Published:** 2023-08-30

**Authors:** Michael Wöltje, Kristin L. Isenberg, Chokri Cherif, Dilbar Aibibu

**Affiliations:** Institute of Textile Machinery and High-Performance Material Technology, Faculty of Mechanical Science and Engineering, TUD Dresden University of Technology, 01069 Dresden, Germany

**Keywords:** *Bombyx mori*, silk fibroin, wet spinning, tensile properties

## Abstract

The wet spinning of fibers from regenerated silk fibroin has long been a research goal. Due to the degradation of the molecular structure of the fibroin protein during the preparation of the regenerated silk fibroin solution, fibroin concentrations with at least 10% protein content are required to achieve sufficient viscosity for wet spinning. In this study, a spinning dope formulation of regenerated silk fibroin is presented that shows a rheological behavior similar to that of native silk fibroin isolated from the glands of *B. mori* silkworm larvae. In addition, we present a wet-spinning process that enables, for the first time, the continuous wet spinning of regenerated silk fibroin with only 4% fibroin protein content into an endless fiber. Furthermore, the tensile strength of these wet-spun regenerated silk fibroin fibers per percentage of fibroin is higher than that of all continuous spinning approaches applied to regenerated and native silk fibroin published so far.

## 1. Introduction

The silkworm *Bombyx mori* produces silk fibers with a high tensile strength of 300–740 MPa and a high elongation at break of 4–26% [1]. During the natural spinning process in the final larval stage, when the silkworm constructs a cocoon so that it can pupate into a silk moth, the spinning dope undergoes chemical and physical changes. The concentration of silk protein solution increases by up to 25% along the glands [2], while, at the same time, the pH decreases [3,4,5], and the cation composition (Ca^2+^ and K^+^) changes [6]. The gradually tapering shape of the spinning duct exerts shear and elongation forces on the silk molecules, promoting their conformational change [7]. In the end, silk fibers are pulled out via the “figure of eight” head movement of the silkworm and then solidify upon exposure to air. This drawing process is an important step in determining the mechanical properties of the silk fiber [8,9,10,11].

Various experimental approaches (e.g., wet and dry spinning) have already been used in attempts to mimic this natural spinning process to produce silk fibers from regenerated silk fibroin (RSF), with some inducing excellent mechanical properties (as extensively reviewed in [12,13]). However, most of these methods do not allow for continuous production, as a post-spinning treatment in a coagulation bath for several hours is often required or the fibers must be manually drawn. Therefore, such methods are not suitable for industrial production and provide only small fiber samples for laboratory use. Only a few wet-spinning approaches have been able to establish a continuous spinning process for RSF. Different combinations of solvents, such as water [14,15,16], ionic liquids [17,18], and 1,1,1,3,3,3-hexafluoroisopropanol (HFIP) [19], and coagulants, such as ammonium sulfate [14,15], methanol [17] or ethanol [18,19], and citrate buffers [16], have been used to spin continuous fibers with different fibroin concentrations ranging from 10% (*w*/*w*) [19] to 13% *w*/*w* [15], 15% *w*/*v* [14], 17% *w*/*w* [17,18], and 32% (*w*/*w*) [16].Recently, Frydrych et al. presented an artificial wet-spinning process for spinning silk fibers from native silk fibroin (NSF). Here, NSF was isolated directly from the silk glands of silkworms and exhibited tensile strength and an elongation capacity comparable to those of natural silk fibers even though there was a fibroin content of only 6.4% (*w*/*w*) in the spinning dope [20]. Assuming that the maintenance of the molecular structure of fibroin was responsible for this result, this, in turn, implies that it is possible to obtain similar mechanical properties using a RSF solution that has a native-like molecular structure.

Therefore, the aim of the present study was to develop a continuous spinning process to spin silk fibers originating from a non-degraded RSF solution that was obtained via a recently published method that involved using gentle degumming via repeated microwave treatment and ZnCl_2_ for dissolution [21]. The mechanical properties of the resulting fibers should correspond to those of natural silk fibers.

## 2. Results

### 2.1. Spinning Dope

**Figure 1:**
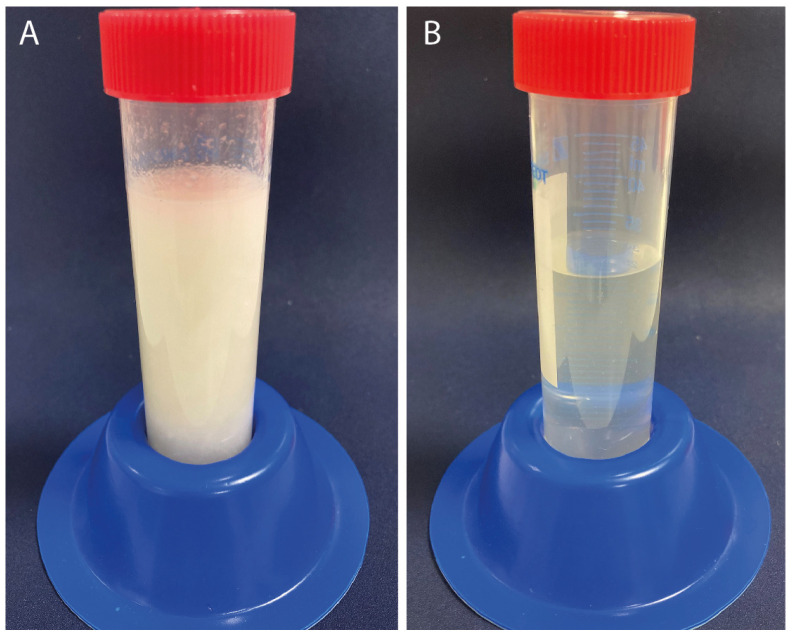
(**A**) Spinning dope obtained from freeze-dried RSF (4% *w*/*v* dissolved in HFIP) 10 min after mixing the two components. (**B**) Spinning dope obtained from freeze-dried RSF (4% *w*/*v* dissolved in HFIP) after overnight incubation at room temperature.

To separate the spinning dopes, freeze-dried RSF was dissolved in HFIP. During dissolution, the sponge-like freeze-dried RSF samples formed a milky suspension, which had a very low viscosity comparable to that of water (Figure 1A). After the incubation of the suspension overnight at room temperature, the RSF was completely dissolved in HFIP. The solution was completely clear and slightly yellowish (Figure 1B) but had a considerably higher viscosity than the original suspension.

**Figure 2:**
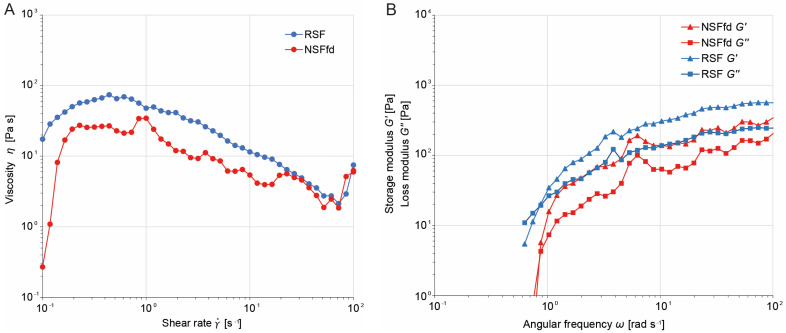
(**A**) Exemplary dynamic viscosity curve for NSFfd (red) and RSF (blue). (**B**) Exemplary oscillatory sweeps for NSFfd (red) and RSF (blue). Elastic modulus (*G*′ triangle) and viscous modulus (*G*″ square) are shown.

In addition, NSF from the silk glands of silkworm larvae was also freeze-dried (NSFfd) and dissolved in HFIP (4% *w*/*v*) for comparison. NSFfd showed the same dissolution behavior, shifting from a white solution to a transparent solution like RSF. However, the NSFfd solution seemed less viscous than the RSF solution. Thus, both NSFfd and RSF were subjected to the same rheological characterization (Figure 2). The viscosity of the RSF and NSFfd solutions decreased with an increasing shear rate, and the solutions exhibited typical shear-thinning behavior with the variation in the shear rate, indicating that both solutions behaved as non-Newtonian fluids with a zero-shear viscosity (*η*_0_) of 17.40 Pa s for RSF and that of 0.27 Pa s for NSFfd (Figure 2A). Furthermore, both silk solutions also showed a slight increase in viscosity at shear rates above 85 s^−1^ (RSF) and above 72 s^−1^ (NSFfd), indicating further shear-induced crystallization. Moreover, oscillatory sweeps of the two silk fibroin solutions revealed an overall increase in both loss (*G*′) and storage (*G*″) moduli. Both solutions only behaved as liquids at low angular frequencies (*G*′ < *G*″), with crossover points (*G*′ = *G*″) at 0.87 rad s^−1^ and estimated relaxation times of 1.15 s for both NSFfd and RSF. At higher frequencies, both solutions exhibited a gel-like behavior (*G*′ *> G*″) (Figure 2B).

**Figure 3:**
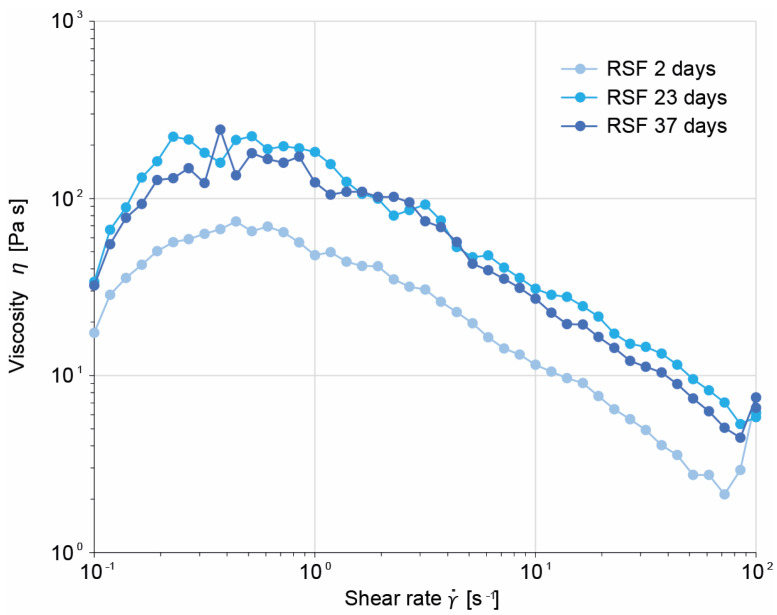
Exemplary dynamic viscosity curves for 4% (*w*/*v*) RSF dissolved in HFIP after storage at room temperature (20 °C) for 2, 23, and 37 days.

In order to analyze the influence of storage and thus HFIP over a longer period, the RSF solution was rheologically examined at different times (2 d, 23 d, and 37 d). As shown in Figure 3, the dynamic viscosity curves of the RSF samples for each storage time show the same progression, indicating that storage did not alter the fibroin structure significantly.

**Figure 4:**
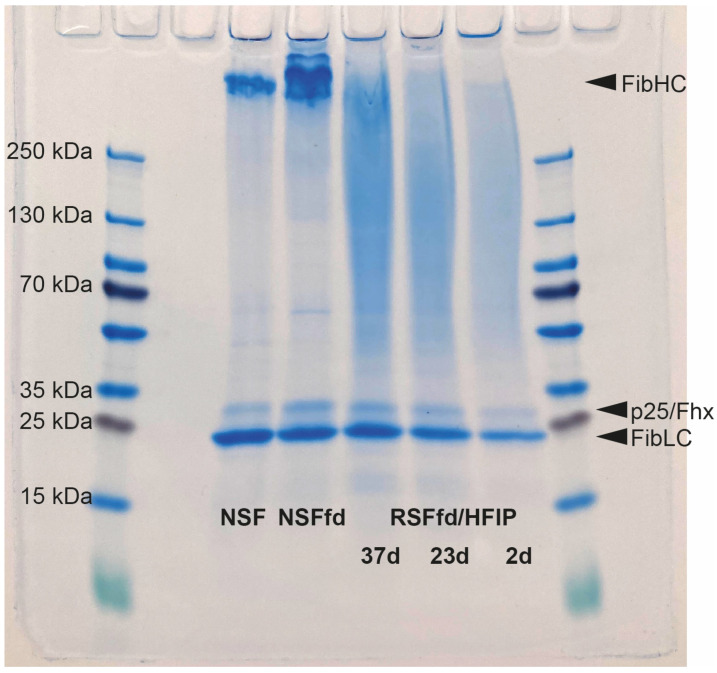
Comparison of molecular mass distribution of NSF and RSF using SDS-PAGE analysis. Films from NSF, freeze-dried NSF dissolved in HFIP (4% (*w*/*v*, NSFfd, stored for 37 d), and freeze-dried RSF dissolved in HFIP (4% (*w*/*v*)) after spending different amounts of time in solution and storage at room temperature (37 d, 23 d, and 2 d, respectively). All samples were dissolved in LiSCN, and 50 μg of each solution was loaded onto a precast gradient polyacrylamide gel (4–20%). PageRuler™ Plus Prestained Ladder (10 to 250 kDa) was used as a molecular mass marker. Arrowheads display molecular mass of the three subunits of silk fibroin (heavy chain fibroin, FibHC, 350 kDa; light chain fibroin, FibLC, 26 kDa; fibrohexamerin/p25, 30 kDa).

To investigate this behavior in greater detail, the influence of the prolonged storage of RSF in HFIP on the molecular mass of silk fibroin molecules was analyzed using SDS-polyacrylamide gel electrophoresis (SDS-PAGE) as shown in Figure 4. Silk films were cast using RSF stored for 2 d, 23 d, and 37 d at room temperature. Films of NFS and of freeze-dried NFS dissolved in HFIP (4% (*w*/*v*); stored for 37 d) were used for comparison. For all the samples, the stained PAGE gel showed three distinct protein bands that can be assigned to the three subunits of the silk fibroin heavy chain (FibHC, 350 kDa), the fibroin light chain (FibLC, 26 kDa), and p25/fibrohexamerin (p25/Fhx, 30 kDa) (Figure 4). This pattern is most clearly visible for the NSF specimens [22], but it is also detectable in the three RSF samples. A comparison of the three RSF samples reveals that the storage time does not affect the integrity of the fibroin molecules. It can also be seen that Freeze drying does not influence the molecular mass of the fibroin molecules (compare NSF and NSFfd).

### 2.2. Wet Spinning and Fiber Morphology

**Figure 5:**
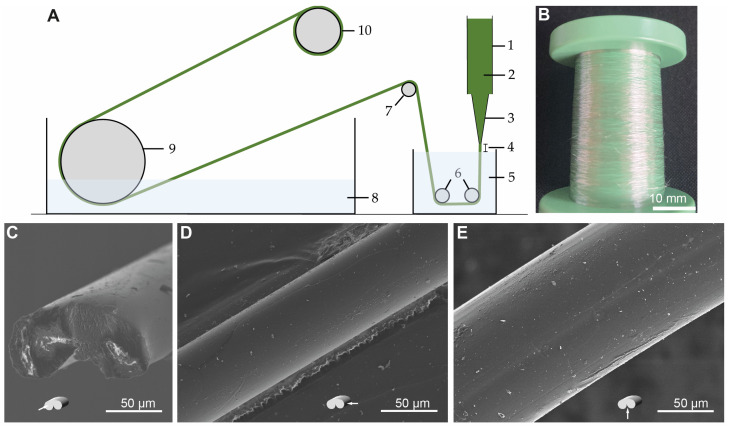
(**A**) Schematic of the custom-made spinning machine for continuous wet spinning: 1—spinning dope storage; 2—spinning dope; 3—spinning nozzle; 4—air gap; 5—coagulation bath; 6—roller; 7—draw roller 1; 8—washing bath; 9—draw roller 2; 10—collecting spool. (**B**) Wet-spun lustrous RSF fiber on a spool. (**C**–**E**) SEM images of ribbon-shaped wet-spun RSF fibers: Cross-section (**C**), side view (**D**), and bottom view of wet-spun RSF fiber (**E**).

A custom-designed and -built wet-spinning machine (Figure 5A) was used to spin monofilament fibers from RSF. A spinning solution of 4% RSF (*w*/*v*) dissolved in HFIP was extruded from a conical dosing needle with an inner diameter of 250 µm (Figure 5A3). The spinning dope was extruded through a 2 mm air gap (Figure 5A4) with a flow rate of 50 µL min^−1^ into an 80% (*v*/*v*) EtOH solution based on the design presented in [19], 0.6 M of ammonium acetate, and a 1% (*w*/*v*) PEG_4000_ coagulation bath (with a pH of 5.3) based on the design presented in [20] at 21 °C (Figure 5A5). Immediately after the RSF spinning dope contacted the coagulation liquid, a clearly visible fiber formed, which was grasped with tweezers. The coagulation distance or the residence time in the coagulation bath was extended using two rollers (Figure 5A6), and the filament was then guided into the washing bath (Figure 5A8) via a godet driven by a servomotor (Figure 5A7). This first godet ensured the initial extraction of the fiber within the coagulation bath (speed: 7.3 mm s^−1^). In the washing bath, the fiber was then guided through the washing bath over a roller (Figure 5A9), which was also driven by a servomotor, and drawn for the second time (at a speed of 18.75 mm s^−1^). From the roller to the spool, the fiber was stretched in a drying section (Figure 5A10) and was drawn for a third time (speed 22.8 mm s^−1^). The final total draw ratio was 3.1. A speed of 1 m min^−1^ was applied during the continuous spinning process. The continuously spun endless fiber (Figure 5B) had a ribbon-like cross section (Figure 5C) with a very smooth surface (Figure 5D,E).

### 2.3. Mechanical Properties of Wet-Spun RSF Fibers

**Figure 6:**
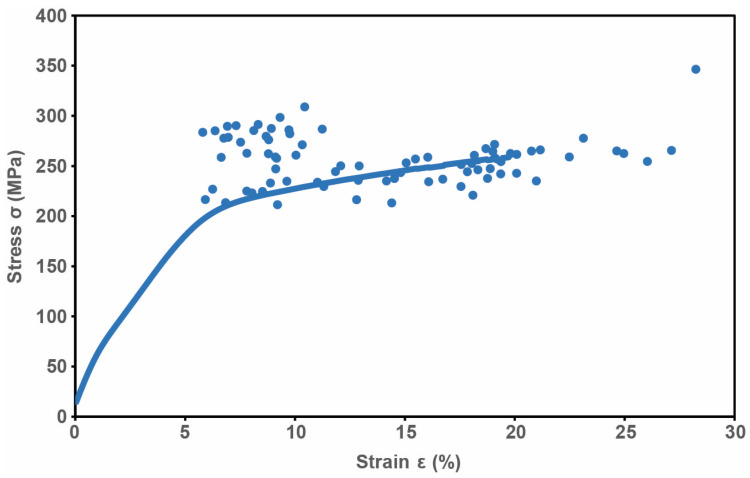
Mechanical properties of RSF fibers. Representative tensile stress–strain curve of fibers wet-spun from freeze-dried RSF (4% *w*/*v*) dissolved in HFIP. Dots denote the breaking points of all specimens tested (n = 80).

The mechanical properties of the wet-spun RSF fibers were investigated. The average tensile strength reached 256.8 ± 13.9 MPa, with an average strain of 13.9 ± 4.7% (Figure 6) and a Young’s modulus of 13.2 ± 1.8 GPa. In this context, the ratio between the amount of fibroin used (4% dry mass) and the tensile strength achieved via its use should be particularly emphasized. With the developed spinning process presented herein, 64.2 MPa per percentage of used dry mass fibroin was achieved.

### 2.4. Secondary Structure Analysis

**Figure 7:**
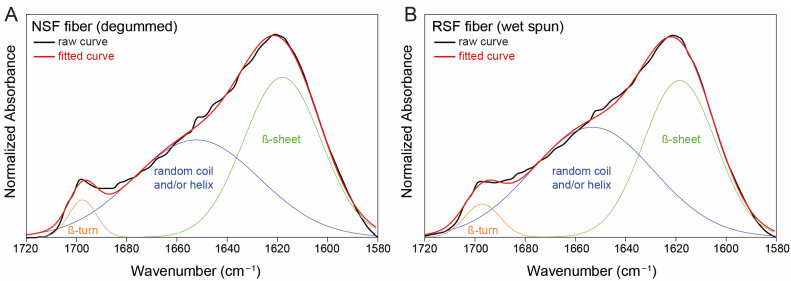
Representative FTIR absorbance spectra of the amide I band (1720 to 1580 cm^−1^) of degummed NSF fibers from *B. mori* cocoons (**A**) and wet-spun fibers from RSF (**B**). The raw curve is shown in black, and the fitted curve is displayed in red. The colored lines show the contribution of the spectra of each type of secondary structure (orange: β-turn; blue: random coil and/or helix; green: β-sheet).

Fourier self-deconvolution of the amide I region was conducted to computationally resolve overlapping lines and quantify the content of the β-sheet, random coil and/or helix, and β-turn to determine the degree of crystallinity. As shown in Figure 7, the degummed NSF fibers and RSF fibers showed similar structural component profiles. The quantification of the content of β-sheets, random coil and/or helix, and β-turns is shown in Table 1, presenting only slight differences between the two types of fibers. An important measure of crystallinity is the β-sheet content, which was 40.0 ± 6.1% in the wet-spun RSF fibers and 44.4 ± 5.6% in the degummed NSF fibers from *B. mori* cocoons.

### 2.5. Cytotoxicity

**Figure 8:**
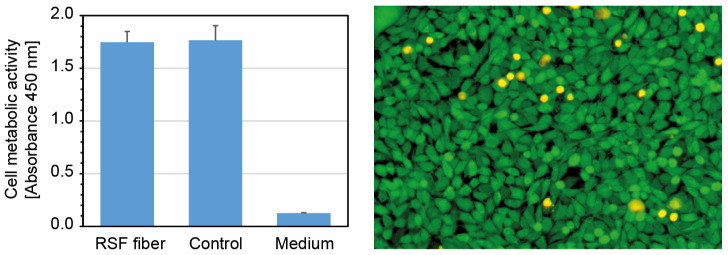
Cytocompatibility of wet-spun RSF fibers: (**Left**): XTT assay for determining cell viability of L929 cells cultivated with extracts from wet-spun RSF fibers for 24 h. The mean cell metabolic activity and standard error of the mean are reported for four independent spinning experiments conducted (at least) in duplicates. (**Right**): Representative fluorescence microscopy image of live–dead staining of L929 cells after 24 h of cultivation with extracts from wet-spun RSF.

Cytocompatibility is a prerequisite for using wet-spun RSF fibers for the development of medical devices or for tissue-engineering applications. Therefore, the viability of cells was investigated. According to [23], the viability of cells after 24 h of incubation with an extract from RSF fibers was analyzed by means of an XTT assay. The cell metabolic activity of the cells incubated with the RSF fiber extract showed only a slight reduction in vitality (3.1%) in comparison to the untreated control cells (Figure 8 left).

In addition, live–dead staining was performed after 24 h of cultivation with RSF extracts (Figure 8 right), showing only very few dead cells (yellow fluorescence), while the majority of the cells remained alive (green fluorescence). Both results show that no substances with cytotoxic effects could be extracted from the wet-spun fibers; thus, RSF fibers do not induce cytotoxic effects on L929 cells.

## 3. Discussion

In 1993, a patent describing a process for making silk fibroin fibers out of regenerated silk was filed. In this process, the cocoon material was degummed via boiling for 90 min in sodium carbonate and “Ivory” soap. The silk fibers were dissolved in lithium thiocyanate hydrate and desalted via dialysis, and the fibroin solution was cast into fibroin films. Then, the fibroin films were dissolved in HFIP, and the obtained fibroin solution was extruded into a methanol bath, leading to the formation of fibers. The patent claims that only a content ranging from at least 5% to a maximum of 25% by weight of fibroin material in HFIP can form a fiber [24]. As recently shown, degumming via the boiling of silk fibers from cocoons in sodium carbonate for 30 min already degrades silk fibroin molecules, and a gentle degumming step is the key to obtaining a non-degraded regenerated fibroin protein solution [21]. From this information, it can be deduced that it should hypothetically be possible to spin a fiber with less than 5% fibroin content using a less-degraded fibroin solution. In order to investigate this hypothesis, 4% (*w*/*v*) freeze-dried RSF isolated via gentle degumming and ZnCl_2_ dissolution was dissolved in HFIP.

There are several results that support this hypothesis: (1) Both the RSF and NSF solutions behaved as non-Newtonian fluids (Figure 2A). This behavior is consistent with previous reports regarding NSF [25,26]. However, such a behavior has never been described for RSF. (2) The 4% (*w*/*v*) NSFfd showed a zero-shear viscosity (*η*_0_) of 0.27 Pa s, which is in good accordance with the findings of Frydrych et al., who reported a viscosity of 0.15 Pa s for 5.1% NSF (*w*/*w*) [20]. However, the viscosity for 4% (*w*/*v*) RSF was 17.4 Pa s, which is the highest viscosity ever measured for a 4% (*w*/*v*) RSF solution. (3) Both silk solutions showed shear-induced crystallization (Figure 2A), as described by Moriya et al. [27], but such behavior has not yet been reported for RSF. In addition, Frydrych et al. [20] described a similar rheological effect in a high-shear-rate regime of NSF. They argued that shear induced sample gelation, which was accompanied by an increase in shear rate as well as a rapid change in normal force, as shown in [28]. Furthermore, a similar effect was reported by Yao et al. [29] after they added silk nanofibers to RSF spinning dopes. They proposed that the nanofiber surfaces act as templates for silk crystallization and thus effectively lower the crystallization activation energy. (4) Both solutions exhibit viscoelasticity with a liquid-like behavior at low frequencies and a gel-like behavior at high frequencies (Figure 2B), as also described by others with respect to NSF [27,30,31,32] but not RSF. This means that at very low angular frequencies, both materials have enough time to respond to cyclic deformation by reorganizing their internal structures and dissipating energy. In this region, the loss modulus (*G*″) is more dominant than the storage modulus (*G*′), meaning that both materials behave more like a viscous fluid. Both materials can flow and dissipate energy efficiently, resulting in a relatively higher G″ value. As the angular frequency increases, NSFfd and RSF have less time to respond to the deformation cycles. This means that they begin to behave more elastically because they do not have sufficient time to flow and dissipate energy. At some point, the storage modulus (*G*′) starts to increase and becomes greater than the loss modulus (*G*″). This frequency is known as the crossover point. Beyond the crossover point, the material has very little time to rearrange its internal structure, and its response becomes predominantly elastic. Normally, as shown for NSF by others [28,32,33], both *G*′ and *G*″ tend to plateau with further increases in angular frequency. This is because the material becomes more resistant to flow and energy dissipation and its response becomes closer to that of a solid. However, in the cited experiments, NSF with a concentration of around 25 wt% was used [2]; thus, a clear plateau was reached at an angular frequency of around 50 to 100 rad s^−1^. In the curve presented herein (Figure 2B), it is evident that there still is a slight increase for *G*′ and *G*″. This probably is due to the low fibroin concentration of only 4% *w*/*v* of NSFfd and RSF used for the oscillatory sweep experiments. In addition, the estimated relaxation time for both NSFfd and RSF was 1.15 s, which is in good accordance with the value of 1.2 s found by Moriya et al. [27], but it contrasts with findings that presented a significantly lower relaxation point for NSF (0.1 to 0.2 s) [28,30]. In contrast, Laity et al. showed that there was a huge variation in relaxation times in their study. They analyzed the oscillatory data of 115 experiments and found very fast (0.055 s), mid-range fast (0.442 s), slow (3.4 s), and very slow (66 s) relaxation times [32]. However, it remains to be noted that the RSF solution used here has a comparable relaxation time with the NSFfd solution. A possible reason for the deviating relaxation time could be that the RSF also contains degraded fibroin molecular chains despite the gentle processing step (Figure 4) and that HFIP, as a solvent, influences molecular interactions, which, ultimately, are also responsible for the rheological behavior.

For the industrial application of a spinning process, it is advantageous when the spinning dope retains its properties over a long period, thus making it possible to spin a yarn with consistent quality. Therefore, the influence of the storage time on the RSF solution was investigated, and it was shown that the storage of the spinning dope over a period of 37 days had no influence on the rheological behavior of the RSF solution (Figure 3). SDS-PAGE analysis also verified that neither freeze drying nor the dissolution of the freeze-dried silk in HFIP negatively affected the spinning solution (Figure 4, NSF vs. NSFfd). In addition, no significant change in the molecular structure of the fibroin in the spinning dope was observed over the study period of 37 days in the SDS-PAGE; therefore, this aspect is not influenced by storage time (Figure 4). Thus, the storage of freeze-dried RSF feedstock and of unused spinning dope at room temperature can be achieved without provoking negative effects on the molecular structure of the RSF and allows for flexible handling during the spinning of RSF fibers. This is a major advantage over previous RSF solutions, for which the silk protein solution changes after some time and eventually becomes solid, even when stored at only 4 °C [34].

A wet-spinning system was developed to validate the hypothesis that a spinning dope made of RSF, which is similar to natural silk in terms of its rheology and molecular structure, can be continuously spun into endless RSF fibers even with fibroin concentrations below 5%. So far, no RSF spinning dope that has native-like properties has been reported or continuously wet-spun into fibers. Here, a continuous fiber was spun using only a 4% (*w*/*v*) native-RSF spinning dope at a speed of 1 m min^−1^. When extruding the RSF dope from a conically tapered dosing needle through a 2 mm air gap into the coagulation bath (Figure 5A), a fine stable fiber formed very quickly, which was then sequentially drawn using three rollers, with a final draw ratio of 3.1, and wound on a spool (Figure 5B).

An air gap approach was followed because it allows for higher take-off speeds and higher deformation of the fiber and thus facilitates a more productive process. The difference between the air gap approach and wet spinning lies in the location where the deformation of the fiber takes place. The deformation of the fiber takes place at the point where the spinning mass can be stretched most easily. This is the case near the end of the dosing needle within the air gap, where the core of the formational fiber is still liquid. In wet spinning, this area lies in the coagulation bath, and the spinning dope immediately interacts with the coagulation bath and forms a solid shell. Through diffusion and coagulation mechanisms, this solid shell moves inwards. Thus, the liquid, easily deformable core is quickly getting smaller. Therefore, the force acting on the fiber during coagulation is negligible in the air-gap-spinning process, while in wet spinning, it contributes significantly to the forces introduced into the fiber. This means that the forces acting on the fiber are lower in the air-gap-spinning process compared to those present in conventional wet spinning [35].

The scanning electron microcopy pictures of the fiber structure showed a ribbon-like structure with a very smooth surface (Figure 5C,D). This is a common effect of wet spinning, and it was also observed by Frydrych et al. in relation to the wet spinning of fibers from 6.4% NSF [20]. One reason for the bean-like shape of the fiber could be that a thin skin forms on the surface of the fibers when the dope enters the coagulation bath, thus encouraging the instantaneous de-mixing of the polymer dope jet. This skin remains as the filament core collapses during diffusion in the coagulation bath, resulting in a bean-shaped fiber. One method of obtaining a more circular fiber diameter is to decrease the water content in the coagulation bath, which encourages the delayed de-mixing of the polymer dope jet. Thus, little to no skin is formed, and the fiber is allowed to contract uniformly [36,37].

Further characterization of the wet-spun RSF fiber via tensile testing showed tensile properties comparable with other published approaches to the continuous wet spinning of RSF (summarized in Table 2). However, all of these used 10 to 32% fibroin content within their spinning dopes. If the amount of fibroin protein spun is correlated with the tensile strength achieved per percentage of fibroin used, the method presented here achieves 64.2 MPa per percentage fibroin protein used. This means that the tensile strength of the silk fiber made from the natural-like RSF was significantly higher than the highest-tensile-strength fiber achieved to date (21.2 MPa per percentage fibroin [17]), which was spun in a continuous process. These results contrast with the very high tensile strength values for wet-spun RSF presented in the literature, such as 1077 MPa [38], 470 MPa [39], 450 MPa [40], or 408 MPa [41]. However, most of these values were obtained using non-continuous processes that have no benefit for commercial technical application.

Nevertheless, a comparison of the wet-spinning process for RSF described herein with the only artificial wet-spinning process for NSF solution described so far shows that NSF has a lower tensile strength per percentage of fibroin (54.7 MPa) when using a 6.4% (*w*/*w*) fibroin concentration [20] than that of native-like RSF (64.2 MPa).

Furthermore, in silk fibroin, the formation of β-sheet structures contributes significantly to its crystallinity. With the development of β-sheet structures and the formation of hydrogen bonds between adjacent chains, these organized regions become stiffer and more stable. This leads to an increase in the overall crystallinity of the silk fibroin material. Thus, the formation of β-sheet structures contributes to the development of organized crystalline regions in the material, as reviewed in [42]. The more β-sheet structures that form, the higher the degree of crystallinity of the silk fibroin. This interplay between molecular organization and secondary structures is a key factor determining the mechanical and functional properties of silk fibroin-based materials. Therefore, the β-sheet content in wet-spun RSF fibers was compared to the β-sheet content of degummed NSF fibers. The determined β-sheet content in the wet-spun RSF fibers, i.e., 40 ± 6%, is only 4% lower than the β-sheet content in the degummed NSF fibers (Figure 7 and Table 1) and is thus in good agreement with the β-sheet content of silk fibroin fibers in the literature, which varies from 28% [43] to 31% [44], 35% [45,46], 42% [47], 50% [48], and 58% [49].

In conclusion, it has been demonstrated for the first time that continuous spinning using a silk solution with a fibroin content of less than 5% is possible. Although the total tensile strength does not yet reach that of natural silk, the tensile strength per percentage of fibroin is higher than that of all spinning approaches for RSF and NSF published so far. Additionally, the wet-spun RSF fibers show a degree of crystallinity similar to that of natural fibers.

Finally, the suitability of the wet-spun RSF fibers for biomedical usage or regenerative medicine was evaluated using indirect cytotoxicity tests. Extracts of the wet-spun RSF fibers were incubated with L929 cells. As defined in ISO 10993-5:2009 [23], a growth inhibition level of more than 30% compared to the untreated control is considered to be a clear cytotoxic effect. As shown in Figure 8, no cytotoxic effect could be detected in either the XTT assay or the live–dead-staining test. Hence, the resulting wet-spun RSF fiber is cytocompatible and thus usable in medical applications.

## 4. Materials and Methods

### 4.1. Materials

Cocoons of the silkworm *Bombyx mori* and 5th instar larvae were provided by the Council for Agricultural Research and Economics, Research Centre for Agriculture and Environment, Sericulture Laboratory, Padova, Italy. Unless otherwise stated, all chemicals utilized in this study were purchased form Carl Roth (Karlsruhe, Germany).

### 4.2. Silk Degumming

For degumming, a recently developed mild procedure that does not damage the molecular structure of fibroin was used [21]. In brief, *Bombyx mori* cocoons were cut into small pieces, and 2 g of these cocoon pieces was heated for 1 min in 125 mL of an aqueous solution of 0.02 M Na_2_CO_3_ (Grüssing, Filsum, Germany) and 0.25% sodium dodecyl sulfate (SDS) in a 250 mL Duran^®^ bottle in a microwave at 800 W. After microwave treatment, the solution containing the silk was left for 10 min at room temperature. Afterwards, the silk fibers were rinsed for 1 min with distilled water. Excess water was removed by squeezing the silk fibers. Then, after 3 additional cycles (microwave heating in fresh degumming solution, a 10 min rest, and washing and squeezing), the degummed silk fibers were dried overnight or directly used for dissolution.

### 4.3. Dissolution of Silk Fibroin Fibers and Desalting

All dissolution experiments were carried out using ZnCl_2_ as a solvent as described in a previous study [21]. In brief, 2.5 g of degummed silk fibers was incubated in 25 mL (10%, *w*/*v*) of 56% (*w*/*w*) aqueous ZnCl_2_ solution at 45 °C in a water bath for 1 h with constant stirring applied. The silk fibroin solution was dialyzed, using a Membra-Cel™ regenerated cellulose membrane with a molecular weight cut-off of 14,000 Dalton against water, for 48 h or until the conductivity was below 10 µS. Dialysis was performed at 4 °C because dialysis at room temperature causes the silk fibroin to gel within the dialysis tubing.

### 4.4. Preparation of Native Silk Fibroin from Silkworm Silk Glands

For the isolation of native silk fibroin, silkworm larvae within the 5th instar were reared on an artificial diet until they entered a resting stage and started emptying their guts. These larvae acquired a translucent appearance and started spinning cocoons. At this stage, the larvae were collected and stored at 4 °C so that they would be anesthetized. The native silk fibroin was isolated according to the protocol reported by Frydrych et al. [20]. Briefly, metal wires were spun across a 50 mL plastic beaker with small incisions within which the wires were placed. The beaker was filled with 10 mM of ammonium acetate extraction buffer solution (pH 7.3 at 10 °C; adjusted with a 1% *v*/*v* ammonia solution). Glands were dissected in a petri dish, washed with extraction buffer to remove detritus, and transferred to a second petri dish prepared as before. Incisions in the anterior and posterior middle gland were made, and the prepared gland was placed on the wire of the dope collector (at the hairpin loop of the posterior middle and middle sections). An additional extraction buffer was added to cover the glands, and Parafilm was placed over the top to prevent solvent loss. The filled beaker was then stored at 4 °C overnight, allowing for gravity-assisted extraction. Then, wires containing the empty silk glands were removed from the beaker, and the upper layer containing buffered sericin was pipetted out, whilst the lower layer of buffered fibroin was filtered at 4 °C under the action of gravity through a fine mesh to remove residual elements of gland epithelia. The filtered solution was then stored at 4 °C until further testing. NSF concentrations were determined by comparing the weight of the dry mass against known wet masses.

### 4.5. Freeze Drying of Silk Fibroin

Desalted silk fibroin samples were first frozen at −20 °C and then freeze-dried using an Alpha 1-2 LDplus freeze-dryer (Martin Christ Gefriertrocknungsanlagen GmbH, Osterode, Germany).

### 4.6. Preparation of Spinning Dope

Spinning dope was prepared via the dissolution of 0.4 g of freeze-dried fibroin material in 10 mL 1,1,1,3,3,3-Hexafluor-2-propanol (HFIP), leading to the acquisition of a spinning dope with 4% (*w*/*v*) fibroin content. After the addition of the HFIP, the turbid suspension was incubated overnight at room temperature. After this time has passed, a yellowish, viscous, transparent liquid had formed, which was then used for wet spinning.

### 4.7. Preparation of Silk Fibroin Films

Silk fibroin films were cast directly from the solution on the surfaces of polystyrene weigh boats and allowed to dry overnight.

### 4.8. SDS-Polyacrylamide Gele Electrophoresis (SDS-PAGE)

The molecular mass distribution of silk fibroin was studied using SDS-PAGE. Silk fibroin films were dissolved in LiSCN (70%) overnight at room temperature at a concentration of 10 µg µL^−1^. For each sample, 30 µg was loaded on a precast vertical 4–20% Tris/Glycine gel (SERVAGel™ TG Prime™) (SERVA, Heidelberg, Germany) under reducing conditions in Tris/Glycine SDS Buffer (Fisher Scientific, Schwerte, Germany). SDS-PAGE was performed at 250 mA with a molecular mass ladder (PageRuler™ Plus Prestained Protein Ladder, 10 to 250 kDa) (Fisher Scientific, Schwerte, Germany); then, the silk fibroin samples were stained with a colloidal Coomassie staining solution (Quick Coomassie^®^ Stain) (SERVA, Heidelberg, Germany).

### 4.9. Rheological Characterization of Spinning Dope

The rheological behavior of NSF and RSF was characterized using a HAAKE MARS III Rheometer (Thermo Fischer Scientific, Waltham, MA, USA). These experiments were conducted using a cone (Ø: 25 mm; angle: 2°) and plate geometry. NSF or RSF solution, in amounts sufficient to completely fill the volume of the sample space, was placed on the plate (fixed), and the cone (mobile) was lowered. Just before the cone touched the silk protein solution, the closing speed was reduced to minimum. Excess solution was allowed to squeeze out from under the cone and was not removed to avoid shear-induced gelation at the edge of the sample space [32]. Dynamic viscoelastic behavior (elastic (storage) and viscous (loss) moduli, *G*′ (*ω*) and *G*″ (*ω*)) were measured as a function of frequency *ω* in the range of 0.623 to 623 rad s^−1^ at 20 °C. Shear viscosity *η* was measured as a function of shear rate *γ*˙ in the range of 10^−1^ to 100 s^−1^.

### 4.10. Wet Spinning

A wet-spinning apparatus was designed and implemented to extrude endless RSF fibers in a continuous spinning process (Figure 5). The RSF spinning dope containing 4% (*w*/*v*) freeze-dried fibroin protein dissolved in HFIP was extruded in a coagulant solution of EtOH (80% (*v*/*v*)), ammonium acetate (0.6 M), and PEG_4000_ (1% (*w*/*v*)) with a pH of 5.3 at room temperature (20 °C). RSF was delivered through a spinning nozzle (with an inner diameter of 250 µm) with a pumping speed of 50 µL min^−1^ and an air gap of 2 mm. After the RSF solution entered the coagulant, a shiny fiber formed that could be transferred to the first draw roller using forceps. The fiber was then transferred to the second roller outside the coagulation bath and guided to a third roller with a large diameter, immersing the fiber into a washing bath containing distilled water. From there, the fiber was wound on a spool. The spinning speed was ca. 1 m min^−1^.

### 4.11. Fiber Characterization

Tensile properties of wet-spun RSF fibers were analyzed using the automatic single-fiber test system FAVIMAT + (Textechno H. Stein GmbH & Co. KG, Mönchengladbach, Germany). Breaking strength (stress), elongation (strain), and Young’s modulus were measured according to [50]. Additionally, 20 mm test specimens (n = 20) with a preload of 12.5 MPa were used to test the fiber from the three independent spinning experiments.

### 4.12. Attenuated Total Reflection Infrared Spectroscopy (ATR-FTIR)

ATR-FTIR spectra of silk fibroin fibers were recorded using a Nicolet 6700 spectrometer (Thermo Fisher Scientific, Waltham, MA, USA). The spectrometer was equipped with a single-bounce diamond ATR accessory (SmartiTR, Thermo Fisher Scientific, Waltham, MA, USA) with a refractive index of 2.4 and an active sample area diameter of 1.5 mm. Spectra of each of the samples were acquired by pressing the fibers against an ATR crystal. All samples were measured in reflection mode. For all measurements, the following parameters were used: (i) resolution of 4 cm^−1^, (ii) spectral range of 650–4000 cm^−1^, (iii) a Norton and Beer apodization window (strong), and (iv) and 64 scans. Deconvolution of amide I band was carried out using the Peak Deconvolution App in OriginPro 2020b (OriginLab Corporation, Northampton, MA, USA). The numbers and positions of peaks were determined from the results of the second-derivatives spectra. The data obtained from the spectra were expressed as means and standard deviations of three independent fiber samples for each sample type.

### 4.13. Scanning Electron Microscopy (SEM)

The morphology of silk fibroin fibers was examined using SEM. Samples of wet-spun fibers were sputter-coated with carbon using a Cressington 108 auto-sputter coater (TESCAN, Dortmund, Germany) and imaged using a digital scanning electron microscope (FESEM) DSM 982 Gemini (Carl Zeiss Microscopy GmbH, Jena, Germany).

### 4.14. In Vitro Cytotoxicity

Possible cytotoxic effects of wet-spun silk fibroin fibers were analyzed using an indirect cell assay, conducted according to EN ISO 10993-5:2009, and the mouse fibroblast cell line L929 (German Collection of Microorganisms and Cell Cultures, DSMZ, Braunschweig, Germany). L929 cells were cultured in Roswell Park Memorial Institute 1640 (RPMI 1640) medium (Lonza, Basel, Switzerland) containing 10% fetal bovine serum (Fisher Scientific, Schwerte, Germany), 2 mM of Glutamax (Lonza, Basel, Switzerland), and 1.0% (*v*/*v*) penicillin/streptomycin (Lonza, Basel, Switzerland).

Before extraction, fibers were disinfected in 70% (*v*/*v*) Ethanol, washed with PBS, and dried overnight under a clean bench. Then, 0.1 g/mL of silk fibers was used for the extraction of possible cytotoxic ingredients and incubated in RMPI 1640 medium, without phenol red, supplemented with 10% fetal bovine serum, 2 mM of Glutamax, and 1.0% (*v*/*v*) penicillin/streptomycin for 24 h at room temperature. Then, cells were incubated with the extraction solution for 24 h in a CO_2_ incubator.

The XTT Cell Proliferation Assay Kit (Serva, Heidelberg, Germany) was used to quantitatively evaluate cell metabolic activity according to the manufacturer’s protocol after 24 h. The electron-coupling and XTT-labeling reagents were thawed and immediately combined in a 1 μL:50 μL ratio. Then, 50 µL of this XTT solution was added to the cell culture wells of the 96-well plate and mixed gently. The absorbance signals of the samples were measured after 2 h of incubation at 37 °C using a Multiskan FC microplate photometer (Fisher Scientific, Schwerte, Germany) at a wavelength of 450 nm and, for background absorbance, at a wavelength of 650 nm. Normalized absorbance values were calculated via the subtraction of background absorbance from signal absorbance. Samples were evaluated, the mean cell metabolic activity and standard error of the mean are reported for four independent samples of wet-spun RSF fibers in at least duplicates.

In addition, after 24 h cultivation of L929 cell with fiber extracts, living and dead cells were visualized after the staining of cells with fluorescein diacetate (FDA) and propidium iodide (PI) (Merck, Darmstadt, Germany). In living cells, FDA is hydrolyzed by intracellular esterases; thus, viable cells will appear fluorescent green when excited with light in the range of 450–490 nm. PI can only enter dead or dying cells. It binds to nucleic acids and causes them to fluoresce red after excitation at 530–585 nm. For live and dead staining, the cell culture medium was replaced by 100 µL of staining solution consisting of 0.8 µg of FDA and 2 µg of PI in serum-free RPMI 1640. Cells were incubated at room temperature for 5 min in the dark. Then, staining solution was removed, cells were washed with PBS, and finally the medium without FCS was added to the samples. Cells were analyzed with a fluorescence microscope (AxioVert.A1, Carl Zeiss, Jena, Germany).

### 4.15. Statistical Evaluation

Statistical analysis was conducted using one-way analysis of variance (ANOVA) followed by Student’s *t*-test (independent, two sided). Differences were considered significant at *p* < 0.05. All data are expressed as means ± standard error of the mean (SEM).

## Figures and Tables

**Table 1:** Comparison of fibroin conformations (β-turn, random coil and/or helix, and β-sheet content (%) of degummed NSF fibers and wet-spun RSF fibers).

Fibroin	NSF	RSF
β-turn	4.9 ± 1.6	4.4 ± 0.8
random coil and/or helix	50.7 ± 4.1	55.6 ± 5.4
β-sheet	44.4 ± 5.6	40.0 ± 6.1

**Table 2:** Comparison of spinning conditions, tensile properties, and tensile strength per fibroin quantity.

Fibroin	Solvent	Coagulant	Draw Ratio	Strength (MPa)	Strain (%)	Young’s Modulus (GPa)	Ø (µm)	Strength Per % Fibroin (MPa)	Ref.
RSF 15 *w*/*v*	H_2_O	(NH_4_)_2_SO_4_	4	260	78.9		13,2	17.3	[14]
RSF 13 *w*/*w*	H_2_O	(NH_4_)_2_SO_4_	4	98	58.9	4.4	25	7.5	[15]
RSF 17 *w*/*w*	NMMO-H_2_O	Methanol	5.3	360	3.9	13.7	32	21.2	[17]
RSF 17 *w*/*w*	NMMO-H_2_O	Ethanol	2.8	127	12.7	5.3	73	10.16	[18]
RSF 10 *w*/*w*	HFIP	Ethanol		102.5	25		49.1	10.3	[19]
RSF 32 *w*/*w*	H_2_O	PEG, Citrate buffer	9	98	52	2.2	71	3.1	[16]
RSF 4 *w*/*v*	HFIP	Ethanol, PEG, CH_3_COONH_4_	3.1	257	13.9	13.2	43.3	64.2	
NSF 6.4 *w*/*w*	CH_3_COONH_4_	PEG, CH_3_COONH_4_	15	350	17.6	13.8	25	54.7	[20]

(NH_4_)_2_SO_4_: Ammonium sulfate; NMMO: N-Methylmorpholine N-Oxide; HFIP: 1,1,1,3,3,3-hexafluoroisopropanol; PEG: Polyethylene glycol; CH_3_COONH_4_: Ammonium acetate. The gray table line highlights the results of presented study.

## Data Availability

The data presented in this study are available on request from the corresponding author.
